# Experimental Scale-Up and Technoeconomic Assessment of Low-Grade Glycerol Purification from Waste-Based Biorefinery

**DOI:** 10.1021/acs.iecr.3c03868

**Published:** 2024-03-11

**Authors:** Taha Attarbachi, Martin Kingsley, Vincenzo Spallina

**Affiliations:** † Department of Chemical Engineering, University of Manchester, M13 9PL Manchester, United Kingdom; ‡ Argent Energy Ltd., CH65 4BF Ellesmere Port, United Kingdom

## Abstract

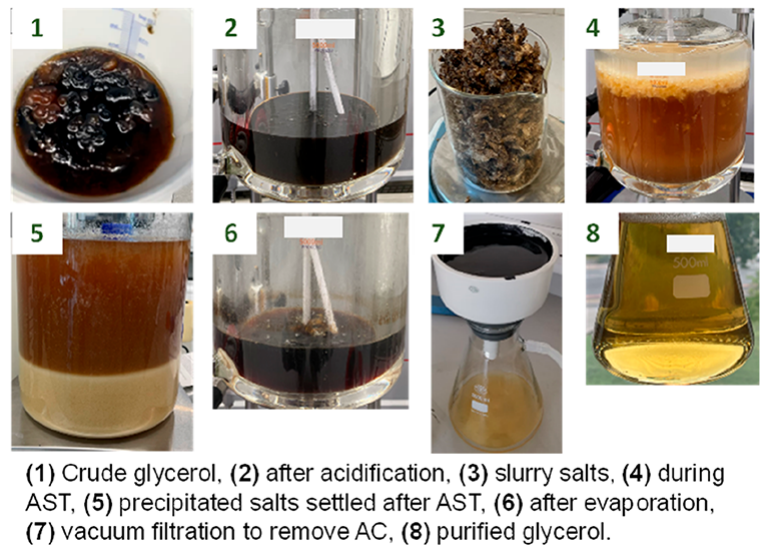

The purification of waste-derived crude glycerol to the 2000 g scale is presented to provide a consolidated proof of concept. Starting from unprecedented low-quality glycerol from a second-generation biodiesel plant, currently disposed of at cost, a series of physiochemical steps are implemented to improve glycerol purity and recovery under relevant conditions. The study is carried out on two samples with initial purities of 38–57 wt % and ash contents of up to 16 wt %. Under the optimal process conditions, glycerol exhibits a remarkable increase to 85 wt % purity while preserving the overall glycerol recovery of the process of up to 71%. Among different purification steps, neutralization contributes to increasing the purity to 69 wt % while the remaining water and methanol evaporation have further increased the purity to >80 wt %. The adsorption step shows the smallest increase in glycerol purity despite it being required to decolorize and deodorize the final product. The developed process is further designed for industrial-scale application using Aspen Plus for a plant size of 1630 kg/h of purified glycerol which could achieve 82 wt % final purity and a maximum recovery of 77%. In addition, the process yields 315 kg/h of salable byproduct salts suitable as fertilizer and an overall CO_2_ emission of 0.70 ton per ton of purified glycerol mainly due to the unrecovered feedstock and solvent combustion. As a result, the proposed process implementation could generate positive revenues with a cost of the final products of €19.2 per ton.

## 1 Introduction

Sustainable biofuels will play a major role next to electric automotives to support the transition toward more carbon-neutral transportation. Renewable Energy Directive EC/2018/2001 mandates a 14% share of renewable energy use in transport by the year 2030.^1^ Advanced biofuels, i.e., biofuels which are based on waste feedstocks, are expected to make up about 3.5% of the market.^2^ Biodiesel plays a major role in this transition, with a market volume of 200 billion USD by 2030, having a CAGR of 8.3% from 2021 to 2023.^3^ As a result, the byproduct crude glycerol^4^ will increase as well up to approximately 6 million tonnes in 2023.^5^

At the same time, biodiesel producers are forced to shift toward alternative feedstocks which are mainly waste-based due to cost and the supply chain.^6^ Exemplary feedstocks are used cooking oil (UCO), animal fats, tallow, FOGs, and POME^7^ considered in second-generation biodiesel refineries, which are more impure and harder to purify (Table 1). Moreover, the high variability of feedstock over time affects glycerol composition, as can be noted in Table 1 (biodiesel producer Argent Energy Ltd.).

**Table 1:** Average Crude Glycerol Composition by Transesterification (First Column)^8^ Followed by Crude Glycerol Composition Derived from Different Locations after Their Post-Treatment

**Component**	**Trans-esterification**	**STANLOW (1) U.K.**	**STANLOW (2) U.K.**	**STANLOW (3) U.K.**	**MOTHERWELL U.K.**	**AMSTERDAM The NL**
Glycerol [wt %]	30–60	36.92	50.89	60.74	45.67	62.59
Ash [wt %]	10–19	12.19	16.11	11.64	8.39	4.54
Water [wt %]	≤10	33.17	7.44	16.92	39.58	17.13
MONG [wt %]	≤40	17.72	25.56	10.7	6.36	15.74

The glycerol-rich phase from the transesterification reaction to produce FAME goes through a sequence of physiochemical treatments aimed at recovering valuable FFA products. Therefore, the final glycerol phase contains additional chemicals and impurities that must be removed. Such glycerol, defined as end of life, is categorized as high-risk (ABP glycerol)^9^ and thus not salable even after deep purification with traditional vacuum distillation; therefore, it is disposed of as biogas feed or fertilizer, and as a result of the bottom stage of the biodiesel value chain, it is discharged at cost (typically >£100 per ton). To handle the large quantity of waste glycerol,^5^ novel purification processes must be developed and scaled up. They should be simple and cheap to implement to reduce costs and waste material for industry (GLAMOUR H2020 project^10^). Alternative and well-established technology such as vacuum distillation^11^ is not suitable for end-of-life glycerol due to the high ash content which could lead to pipe clogging and periodic shutdowns for maintenance. New processes are looking at crude glycerol purification by different means such as physiochemical treatments,^12−14^ ion exchange,^15^ and membrane distillation.^16^ However, research conducted in this field mainly relies on the use of nonindustrial or nonwaste-based crude glycerol. For example, Pott et al.^17^ have purified crude glycerol derived from canola oil. Dmitriev et al.^18^ have used crude glycerol from an industrial producer (OAO Mogilevkhimvolokno in Belarus) which is utilizing rapeseed oil with very low ash content (3.8 wt %). Nanda et al.^19^ sourced their glycerol from an industrial biodiesel refinery with low ash content as well (5.6 wt %). Chen et al.^20^ have used UCO-derived crude glycerol (<4% ash and less heterogeneous MONG). A more extensive review of the physiochemical treatment for glycerol purification is reported in a previous study by the authors.^5^

Industrial process design assessments, supported by experimental evidence of the purification steps, are not available in the literature. Oliveira et al.^21^ and Braga et al.^22^ have both simulated the purification of crude glycerol using vacuum distillation with a slight physiochemical pretreatment to achieve a purity of 99.7%. Despite the appreciable attempt to assess the process performance on the industrial scale, Braga et al.^22^ have assumed a starting purity of 80 wt % with an ash content of 3 wt %, and Oliveira et al.^21^ have used an initial purity of 50 wt % not including any ash content and a very high methanol content (35 wt %). Another study from Xiao et al.^23^ simulated a complex physiochemical purification process to reach purities of up to 94 wt % when neglecting the ash removal step. Hence, while providing a good indication of energy requirements and cost, these studies are not applicable to the example of a more severe feedstock as considered in this study (Table 1).

Given the lack of research and industrial understanding in the field of biobased purification processes, this article provides the first attempt to assess the impact of unprecedented contaminated low-quality glycerol purification and scale up the developed process. Compared to existing research and previous studies reported in the literature, this work extends the relevance of physiochemical treatment by the experimental proof of concept of different waste-derived glycerol samples, and the relevant conditions and results obtained on the laboratory scale have been implemented in a process simulation flowsheet to assess an industrial-scale plant that could use multiple glycerol sources with different properties, representative of next-generation biorefineries. The resulting technoeconomic assessment has been validated by an industrial operator, thus providing a solid background for similar studies in the area of waste purification in the oleochemical industry. This study follows up on a previous work of the authors to optimize the purification performance on 100 g batches using a rigorous custom design of the experimental approach with the relevant response surface methodology^24^ which is now scaled up to 2000 g. Compared to our previous work, this study addresses relevant industrial challenges such as the use of suitable chemicals that could generate valuable byproducts (e.g., fertilizers) and use or recycle existing materials available in the biodiesel supply chain. Hence, this work is informative for industry to provide realistic technoeconomic key performance indicators and develop a new route for biomass valorization. A close study is therefore carried out to assess the impact on glycerol recovery, ash removal rate, and the use of chemicals as well as try different configurations/adjustments of the purification route due to the different composition of the starting material and associated technoeconomics.

## 2 Materials and Methods

### 2.1 Materials

#### 2.1.1 Crude Glycerol

The crude glycerol samples were obtained from a biodiesel refinery in Stanlow (England, U.K.). Crude glycerol compositions are reported in Table 2 based on titrimetry and HPLC characterization to appreciate the differences. The biodiesel refinery in Stanlow exclusively uses waste feedstocks based on animal fats, UCO, tallow, or FOGs (based on the market availability). Unlike industrially derived crude glycerol reported in other work (starting from a pH of 9–11 after transesterification), the samples tested in this study are treated downstream in the STANLOW plant. The glycerol pH is first reduced to about 3 (from the original 9–11) in an acidulation step followed by a tricanter which separates the three generated phases FFA, glycerol, and precipitated salt. The FFA layer is then redirected into the biodiesel refinery, and the precipitated salt is stored for other purposes. The glycerol phase is then neutralized and settled to remove additional FFA which was generated during the neutralization. Afterward, the treated glycerol is distilled in a column to recover methanol. The dewatering of the crude glycerol phase is then again followed by a tricanter which separates the three phases: the MONG layer, salt layer, and glycerol. Due to the significant post-treatments, the final glycerol contains all impurities which cannot be recovered, in particular, ashes (>12–16 wt %), and it is sent for disposal at cost (€150 per ton).

**Table 2:** “End of Life” Glycerol Composition Provided by Argent Energy Ltd. at Stanlow, U.K.

	**STANLOW (1)**		**STANLOW (2)**	
**Inlet Composition**	**HPLC**	**TITRIMETRY**	**HPLC**	**TITRIMETRY**
Glycerol [wt %]	56.82	56.87	44.23	38.34
Glycerol (dry) [wt %]	61.39	61.44	66.18	57.40
Ash [wt %]	16.11	16.11	12.19	12.19
Water [wt %]	7.44	7.44	33.17	33.17
MONG [wt %]	19.63	19.58	10.41	16.30

**Figure 1:**
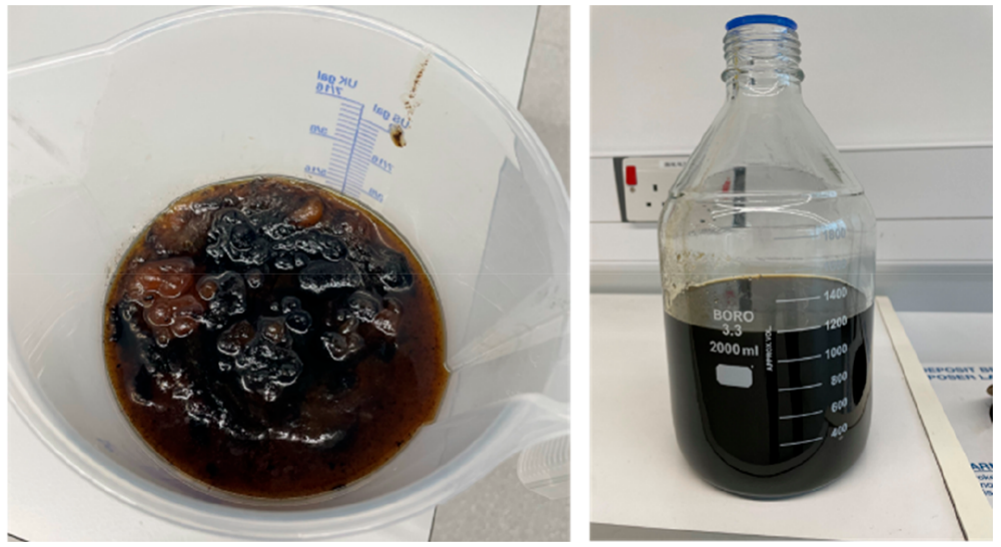
Crude glycerol samples from Argent Energy’s Stanlow refinery. Left STANLOW (1); right, STANLOW (2).

The results for the glycerol purity are given for the HPLC results as well as for the titrimetric method. The high deviation comes mainly from the fact that the HPLC uses the safeguard that removes matter prior to reaching the analysis, including some glycerol as can be seen in the table. The differences in the composition (in particular, the high water/moisture content in STANLOW (2)) have also influenced the appearance of the crude glycerol as shown in Figure 1.

STANLOW (1) shows visible chunks of semisolid MONG. In general, the high water content of STANLOW (2) leads to a higher solubility of hydrophilic MONG and ash content in the mixture and is less viscous than STANLOW (1), which is something between an emulsion and a clear two-phase mixture. An in-house GC–MS analysis has been reported in (Table 3) which gives an overview of approximately 50% of the MONG type of components present in the crude glycerol; the other 50% of MONG remain unidentified. These components are fatty acids, esters, soaps, FAME, and even unusual chemicals such as 4-me-phenol, 4-hydroxy-2-pentenoic acid, and hydrocinnamic acid, leading to a high degree of impurities in the crude glycerol and high volatility in the composition.

**Table 3:** GCMS MONG Analysis of a Sample, Conducted by Argent Energy Ltd.

Fraction	wt %
Short-chain acids	23.1
Long-chain acids	3.8
FAME	16.9
Other	9.4

Other chemicals used in the purification process are given in Table 4.

**Table 4:** Chemicals Was Used for the Experimental Runs and Analytical Measurements

**Purpose**	**Chemical**	**Supplier**	**Product code**	**Grade**
Experimental	Phosphoric acid	ACROS Organics	201140010	85% aqueous for analysis
Experimental	Methanol	VWR	20903.461	TECHNICAL ≥98.5%
Experimental	Potassium hydroxide	Honeywell Fluka	019-002-00-8	ACS reagent; >85% solid
Experimental	Powdered activated carbon	CHEMVIRON	WPS260-90	n/a
Experimental	Propan-2-ol	Fisher Chemicals	P/7500/17	Analytical reagent grade, >99.8%
Analytical	Hydranal Coulomat AG	Honeywell Fluka	34836	n/a
Analytical	Sodium hydroxide	Honeywell Fluka	7139500	0.1 N
Analytical	Sodium metaperiodate	Supelco, EMSURE	1.06597.1000	ACS reagent, for analysis
Analytical	Sulfuric acid	Supelco, TITRIPUR	1.09074.1000	Titripur, 0.1 N
Analytical	Ethylene glycol	Fisher Chemicals	BP230-1	>99%
Analytical	Bromothymol blue	Sigma-Aldrich	114413	ACS reagent, 95%

#### 2.1.2 Experimental Setup

**Figure 2:**
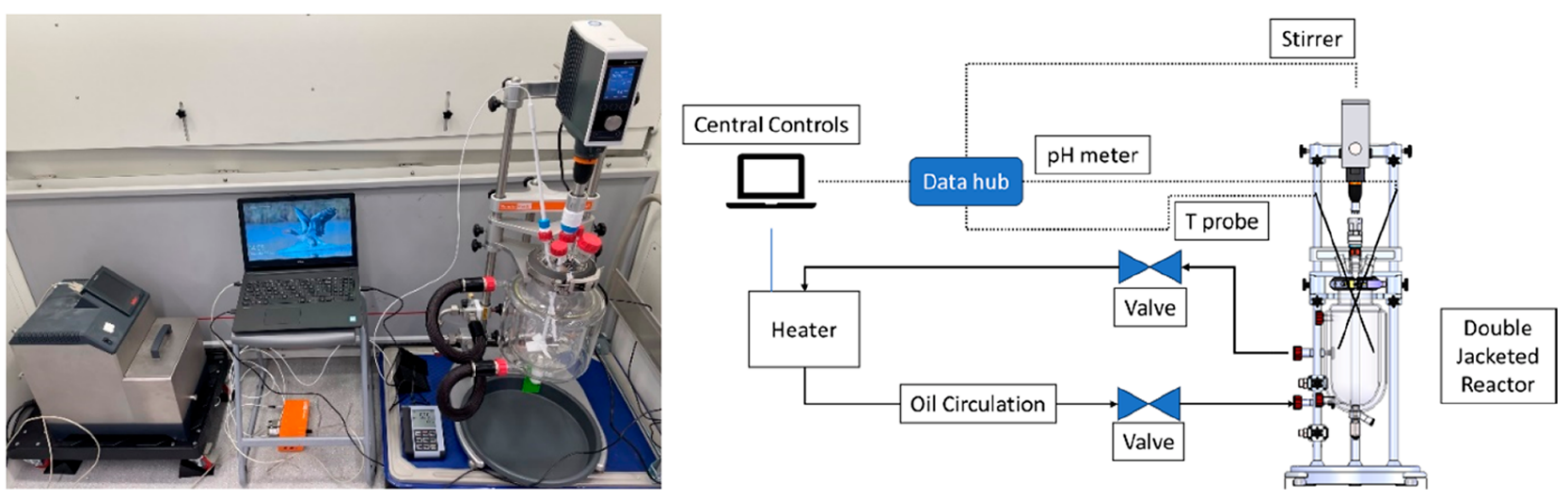
Rig setup consisting of the 5 L batch reactor, heater, and laptop.

A 5 L glass batch reactor from Radleys was used to perform the experiments. The entire rig consisting of the reactor, stirrer, heater, instrumentation, and controls can be seen in Figure 2.

The glass reactor consists of a double jacket which can be filled with silicon oil for heating purposes, loaded at the top, and emptied via a valve at the bottom, which enables easy decantation, e.g., after separation. Furthermore, it has four round openings at the top which can be used for inserting instrumentation (such as a temperature probe, pH probe, etc.) or for the addition of chemicals. The stirrer and temperature probe are made of PTFE material. The stirrer is driven by a Heidolph Hei-TORQUE Ultimate drive. The heater is a ministat 240 provided by Huber that heats the silicon oil while pumping it through the double jacket of the reactor. Customized Radleys AVA software is used to control the rig. The pH value inside the reactor is controlled by a Knick Portavo 904 pH meter in combination with a Hamilton Polilyte Plus H VP 425 pt1000 pH probe.

### 2.2 Purification of Crude Glycerol

**Figure 3:**
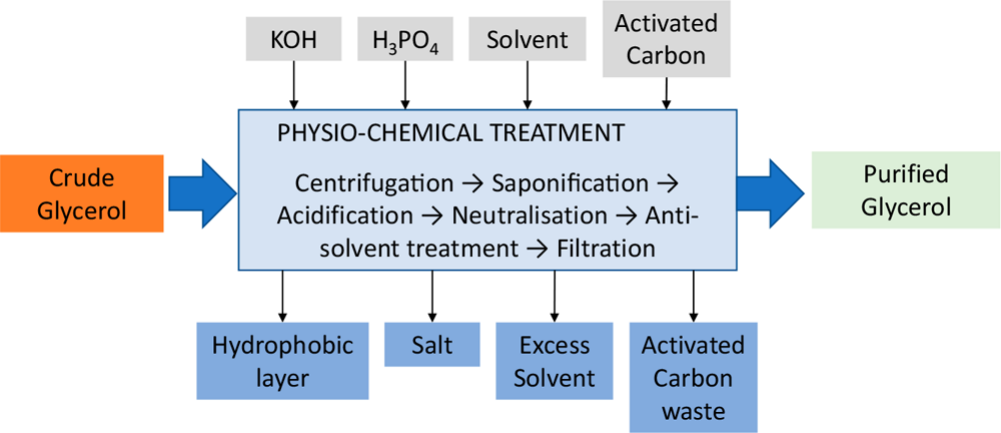
Generic layout of the purification route considered.

The purification route has been optimized in previous work and presented in Figure 3. The general experimental procedure consists of multiple physiochemical steps starting with acid–base treatments such as saponification and acidification, followed by overnight separation, vacuum filtration, neutralization, antisolvent treatment, vacuum filtration, evaporation (until the boiling stopped and just minor bubbles were visible), and activated carbon treatment followed by final vacuum filtration.

In this work, the experiments have always been conducted using 2000 g of crude glycerol. Prior to use, the canister containing the crude glycerol was thoroughly mixed to ensure homogeneity. Depending on the feedstock, the process starts with a separation step by stirring the material for 2 h at 2000 rpm and 80 °C, followed by an overnight separation. Three layers are distinguished: (1) the middle layer emulsion of polar components such as glycerol, water, and polar impurities, (2) the top hydrophobic organic (MONG) layer, and (3) a bottom solid layer. However, not every crude glycerol feedstock exhibited this formation. The glycerol-rich layer (1) is separated by decanting it slowly. Afterward, the glycerol layer was saponified with 12.5 M KOH to a pH of 8 for 1 h at 200 rpm and 60 °C, followed by acidification with 85% H_3_PO_4_ to a pH of 6 for 1 h at 200 rpm at 60 °C. The mixture is again left for overnight separation. Large amounts of salts precipitate overnight, which are removed by vacuum filtration. In the next step, the mixture is neutralized with the same base for 60 min at ambient temperature and 200 rpm. Afterward, methanol is added in a solvent-to-glycerol volume ratio of 3:1 to induce the antisolvent effect and achieve further precipitation of salts. Although isopropanol (IPA) induces the best antisolvent effect,^25^ methanol was used for these experiments. This decision depends on the large availability of methanol in a biodiesel plant. In addition, methanol is cheaper compared to IPA, it does not generate an azeotrope with H_2_O which would result in the accumulation of a water/IPA layer with salts, IPA consumption, and additional cost for purification. In addition, spent methanol from the biodiesel synthesis plant could be used for this step before sending it to final recovery. The precipitated salts are again removed via vacuum filtration. The mixture is then evaporated for 2 h at atmospheric pressure with a maximum temperature of 138 °C to remove methanol and reduce moisture in the glycerol phase. The temperature range of 138–140 °C has been selected to avoid glycerol decomposition and reduce the moisture content according to the binary water–glycerol diagram obtained by Aspen Properties V12.1 at atmospheric pressure. In the last step, pulverized activated carbon is added at a concentration of 50 g/L to decolorize and deodorize the mixture. The activated carbon particles are removed as a cake during the last step via vacuum filtration.

Two key performance indicators are used to assess the purification route, glycerol recovery, and ash removal rate according to eqs 1 and 2, respectively, along with the glycerol purity (wt %).
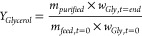
1
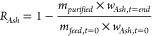
2

### 2.3 Analytical Methods

#### 2.3.1 Glycerol, Ash, Water, and MONG Analysis

The quantification of the glycerol component has been conducted via British Standard BS 5711-3:1979. This method uses titration in which glycerol reacts with sodium periodate (NaIO_4_) in acid aqueous solution to produce formaldehyde and formic acid and is used to quantify the glycerol content. The ash content is analyzed via a gravimetric method according to British Standard BS 5711-6:1979, where 10 g of the sample is poured into a crucible which is first heated via a Bunsen burner to remove organic volatiles. Afterward, the crucible is transferred into a 750 °C furnace (Nabertherm P300) and left for 10 min to remove any residual organic content as recommended in the BS 5711-6-1979 procedure. The residual materials, mainly ashes, are placed in a desiccator for 15 min to remove any excess moisture. Subsequently, the mass difference is measured compared with the initial sample weight to determine the ash content. The water content is measured according to the British Standard BS 5711:8-1979 which utilizes a Karl Fischer titrator (Metrohm 899 coulometer). The MONG content is calculated via eq 3 as a difference from all other components.
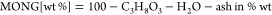
3

#### 2.3.2 HPLC Analysis

The glycerol content in the sample is analyzed with HPLC-RID to validate the accuracy of the titration analysis due to the presence of existing alcohols in the MONG. For the analysis, an HPLC (Thermo Fisher Scientific Ultimate 3000) with an RID was used with an ROA-organic acid with safeguard (Phenomenex 00H-0138-K0, Phenomenex Carbo-H 4 × 3.0 mm ID), with the mobile phase being 1 wt % formic acid solution at a flow rate of 0.6 mL/min with the temperatures of the column and RID being at 60 and 55 °C, respectively.^26^ The volume injected into the HPLC per sample vial was chosen to be 10 μL.^26^ Given the higher accuracy, the discussion of glycerol purity in this work is generally referred to as HPLC analysis unless specified differently.

### 2.4 Process Simulation

Besides the experimental testing of the 2000 g batch size, the process assessment at the industrial scale has been developed and simulated using Aspen Plus V12.1. The two research tasks are linked since the same operating conditions are used, thus providing a realistic quantification of the process performance. More specifically, the ratio among acid (H_3_PO_4_), base (KOH), antisolvent (MeOH), and crude glycerol has been kept the same, and the operating conditions during reactions were also implemented according to the experimental campaign to derive meaningful M&H balances of the integrated process. It should be noted that given the results presented in Section 3.2, the process developed can treat any type of glycerol without providing substantial differences in the final composition but only in the recovery (Figure 11). To provide a more conservative approach, the inlet composition has been assumed to be similar to STANLOW (1) provided in Table 5 due to the fact of the higher ash content (16 wt %), which is more demanding in terms of separation. The ash content has been modeled as a mixture of sodium, phosphorus, potassium, and sulfuric ions, while the MONG content has been modeled as a mixture of different short- (acetic acid) and long-chained fatty acids (*n*-octanoic acid) and soaps (potassium-propionate), short- (methyl-acetate) and long-chained esters (methyl-oleate) in the form of FAME, and glycerides in the form of trilaurin. To account for all components formed during the sequence of the physiochemical process, other ions have been added to the system which can be identified in the Supporting Information where the complete M&H balance of the final process is presented. The property method used for modeling the process was ELECNRTL due to the existence of different ionic species and in agreement with Xiao et al.^23^ and Braga et al.^22^ The reactors have been modeled as RStoics as no kinetic information was available for the process. Generally, the process has been modeled according to the experimental results (purity, recovery, and use of chemicals) which were obtained in this study. The plant size has been decided by assuming that three biodiesel refineries would be clustered to treat the whole amount of glycerol produced, approximately 67 tonnes of crude glycerol per day as in the case of the existing production capacity of Argent Energy in their biodiesel plants in western Europe.

**Table 5:** Inlet Mass Flow and Composition of Crude Glycerol

**Stream Name**	**Units**	**Crude Glycerol**	
**Mass flows**	**kg/h**	**2829.16**	**Mass fractions [−]**
**GLYCEROL**	**kg/h**	**1750.00**	**0.619**
**WATER**	**kg/h**	**209.77**	**0.074**
**MONG**	**kg/h**	**392.00**	**0.139**
**Ash**	**kg/h**	**475.96**	**0.168**
*TAG*	*kg/h*	*28.0*	*0.010*
*K*^*+*^	*kg/h*	*82.1*	*0.029*
*KH*_2_*PO*_4_	*kg/h*	*140.0*	*0.049*
*H*_2_*PO*_4_^*–*^	*kg/h*	*0.00*	*0.000*
*OH*^*–*^	*kg/h*	*0.21*	*0.000*
*HPO*_4_^*2–*^	*kg/h*	*1.21*	*0.000*
*PO*_4_^*3–*^	*kg/h*	*93.45*	*0.033*
*SO*_4_^*2–*^	*kg/h*	*94.64*	*0.033*
*Na*^*+*^	*kg/h*	*65.72*	*0.023*
*L-FAME*	*kg/h*	*56*	*0.020*
*L-FFA*	*kg/h*	*28*	*0.010*
*SS-SOAP*	*kg/h*	*28*	*0.010*
*SS-FAME*	*kg/h*	*56*	*0.020*
*SS-FFA*	*kg/h*	*196*	*0.069*

The CAPEX and OPEX calculations have been prepared according to Towler et al.^27^ The equipment costs have been calculated using eq 4, where *a* and *b* are cost constants, *S* is the size parameter which depends on the equipment, *n* is the exponent for a specific piece of equipment, and *C_e_* is the purchased equipment cost on a U.S. Gulf Coast basis (Jan 2010, CEPCI = 532.9). The values for the constants are taken out from Towler et al.^27^ and the cost index is based on year 2022.^28^

Once the equipment costs have been calculated, a factorial method is used to estimate the project fixed capital costs *C* (or installation costs associated with a specific piece of equipment). The values for the factors can be viewed in Towler et al.^27^ The sum of this is defined as ISBL (inside battery limits).

Based on the ISBL costs, the costs for offsites (OS), design and engineering (D&E), and contingency (X) are summed to obtain the total fixed capital cost according to eq 6 and adjusted for inflation (eq 7) and location (eq 8). It is assumed that glycerol purification will take place in The Netherlands.

Obtaining the final total investment required an additional 5% working capital.^27^
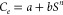
4
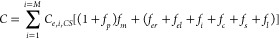
5
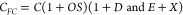
6
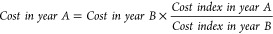
7
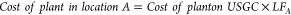
8

The OPEX costs are calculated via Towler et al.^27^ as well by dividing them into variable and fixed costs. The variable costs can be found in Table 6.

**Table 6:** Price of the Material Streams and Utilities

**Raw Material**	**Price per ton [€/t]**
Crude glycerol	–150^a^

**Byproduct**	**Price per ton [€/t]**
Fertilizer	–145.65^29^

**Utilities**	**Price per kW [€/kWh]**
Electricity [€/kWh]	0.1762^30^
Heat [€/kg]	0.01113^31^

**Consumables**	**Price per ton [€/t]**
12.5 KOH	460^32^
85% H_3_PO_4_	1100^33^
Methanol	395^34^
Activated carbon	500^35^
Water	0.45^27^

a Cost of waste glycerol disposal as provided by Argent Energy.

The fixed cost of production is summarized in Table 7. The annual capital charge (ACC) has been calculated using an interest rate of 4%,and a lifetime of 25 years has been assumed for the plant due to its simplicity.

**Table 7:** Fixed OPEX Cost Calculation Based on Towler et al.^27^

**Description**	**Share**	**Share of**
Supervision	25%	Operating labor
Direct salary overhead	40%	Operating labor
Maintenance	3%	ISBL
Property taxes	1%	ISBL+OSBL
Insurance	1%	ISBL+OSBL
Rent of land	1%	ISBL+OSBL

**General Plant Overhead**	**Share**	**Share of**
General and administrative costs	65%	Operating labor
Allocated environmental charges	1%	ISBL+OSBL

The implementation of the cost model is provided as Supporting Information.

## 3 Results and Discussion

### 3.1 Experimental Proof of Concept

The use of different feedstocks implies few modifications. As STANLOW (2) contains 33 wt % moisture, an additional evaporation step has been added to the process to avoid the heavy use of solvent during the antisolvent treatment step. This step is not necessary in the case of <10 wt %. On the other side, STANLOW (1) contains >25 wt % of visible chunks of MONG which accumulate at the top of the mixture. This is because some triglycerides do not convert entirely to FAME during transesterification and some FFA do not convert during esterification, leaving diglycerides/monoglycerides with different fatty acids in the mixture. This type of MONG content, visible in Figure 1, is removed via centrifugation. Three different experiments have been conducted in this work: experiments (1.1) and (1.2) using feedstock STANLOW (1) and experiment (2) using feedstock STANLOW (2).

#### 3.1.1 Characterization of Feedstock STANLOW (1)

**Figure 4:**
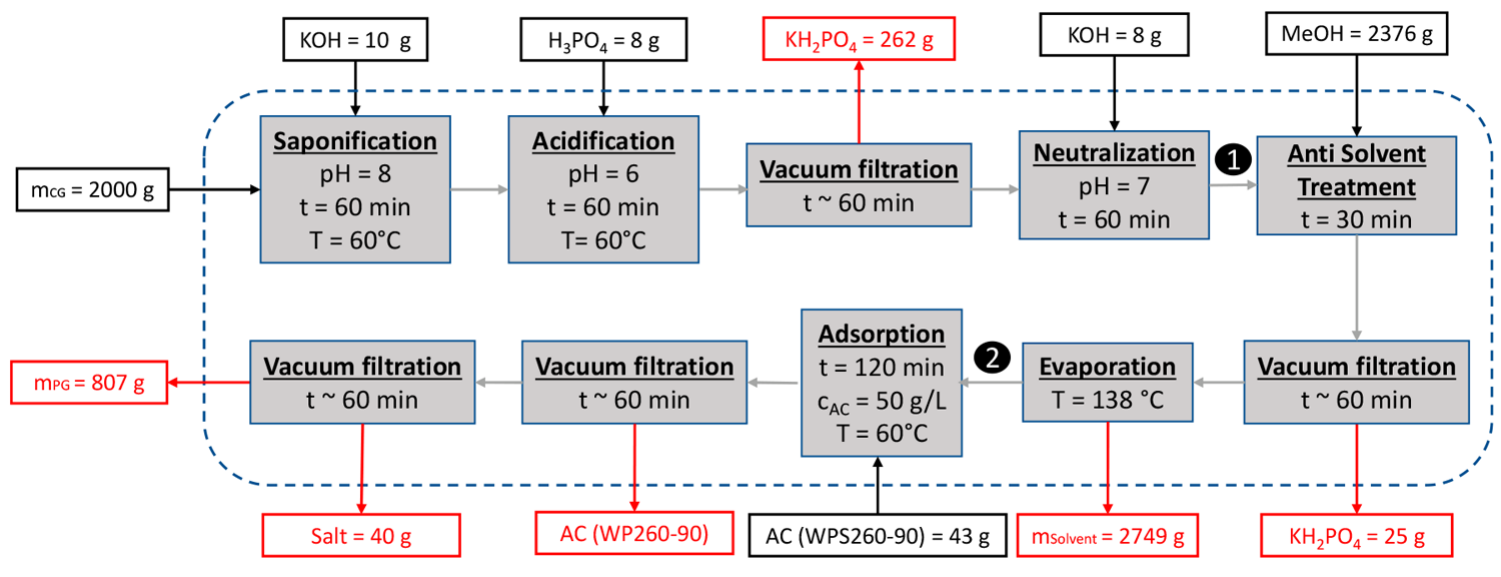
Run 1.1. Purification Route for STANLOW (1). The black dots denote a sample analysis at this specific point, including detailed mass balance and compositions of crude and purified glycerol.

The resulting flowsheet for run 1.1 using STANLOW (1) can be seen in Figure 4.

The purification consists of 9 steps: saponification, acidification, vacuum filtration, neutralization, antisolvent treatment, vacuum filtration, evaporation, adsorption, and vacuum filtration. The first 3 steps are generally used to reduce the MONG content and remove a significant amount of salts present in the solution. The saponification with KOH is used to convert parts of the MONG components, mainly unreacted mono-, di-, and triglycerides, to glycerol and soaps and to convert free fatty acids and fatty acid methyl esters to saponified fatty acids (SFA) (also fatty acid salts or soaps) according to eq 9, eq 10, and eq 11 using a strong basic agent such as potassium hydroxide.
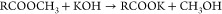
9
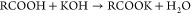
10
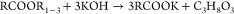
11This reaction is followed by acidification of the mixture by an acid. The literature and previous experiments have shown that phosphoric acid is the best acidification agent.^13^ The soaps are generally converted to fatty acids according to eq 12, and any acid present in the mixture is eliminated via the neutralization reaction (eq 13). The solution is then left overnight to precipitate the salt.
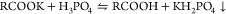
12
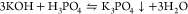
13

In some cases, the liquid phase splits into a middle glycerol layer, a top fatty acid layer, and a bottom salt layer. However, a top fatty acid layer could not be detected because a large amount of FFA had already been removed in the biodiesel plant before sampling the glycerol. The precipitated salts (slurry) are removed via vacuum filtration. The subsequent antisolvent treatment with methanol yields more salts which are removed by vacuum filtration, followed by evaporating the solvent and residual moisture at 138 °C. Finally, the mixture is discolored and deodorized by activated carbon. The activated carbon is removed from the mixture by vacuum filtration. Additional salt may still be formed in the presence of residual chemicals and thus precipitate. The composition of glycerol after neutralization (point 1), after evaporation (point 2), and at the end of the purification process can be seen in Table 8.

**Table 8:** Composition of the mixture at both sample points and of the final sample for Run 1.1

**Composition**	**Initial**	**Point 1**	**Point 2**	**Purified Glycerol**
Glycerol [wt %]	50.89	69.37	80.98	82.02
Ash [wt %]	16.11	10.25	8.31	8.63
Water [wt %]	7.44	8.75	4.28	4.43
MONG [wt %]	25.56	11.63	6.43	4.92

Sample point 1 proves that a simple physiochemical treatment at nonextreme pH points is effective and increases the purity by approximately 18.48 wt % (from the original 50.9 wt %) after some triglycerides are split into soaps and glycerol. Furthermore, the reduction of the ash has been significantly reduced by approximately 6 wt % (from 16.11 wt %). After the salts were dissolved in water, a pH of 4.86 was shown which proves that most of the precipitated salts are KH_2_PO_4_ as in eq 12. The MONG content is reduced by 54% compared to the inlet content.

**Figure 5:**
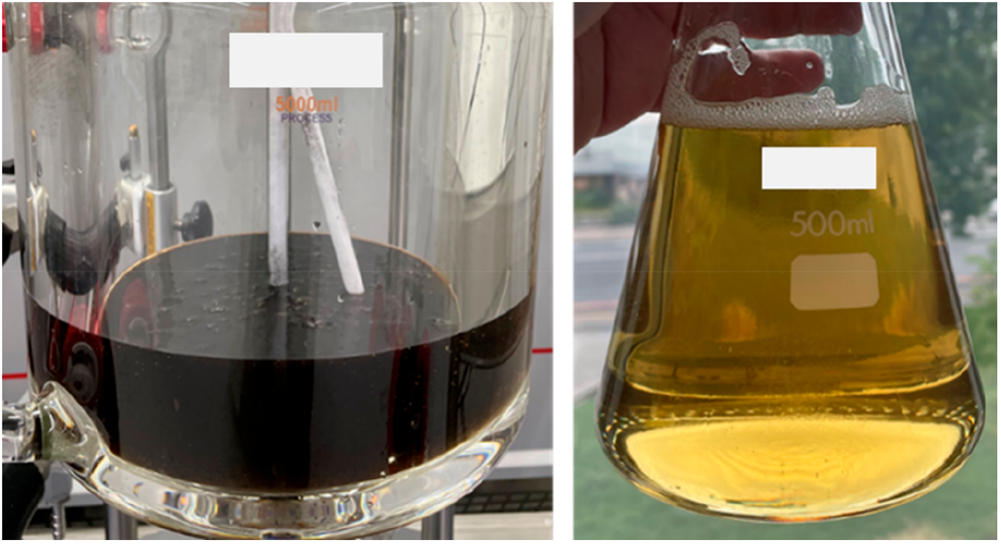
Crude glycerol color (left). Purified glycerol color (right).

Sample point 2 proves that the activated carbon step does not yield a significant increase in the purity. However, a significant decolorization of dark brown, almost black, to light yellow is visible (Figure 5), along with an improvement in the odor as certain organic components are removed by the activated carbon. The evaporation of excess moisture and methanol as well as organics such as short-chain fatty acids at 138–140 °C is beneficial as it increases the glycerol purity by an additional 12 wt % (Table 3).

**Figure 6:**
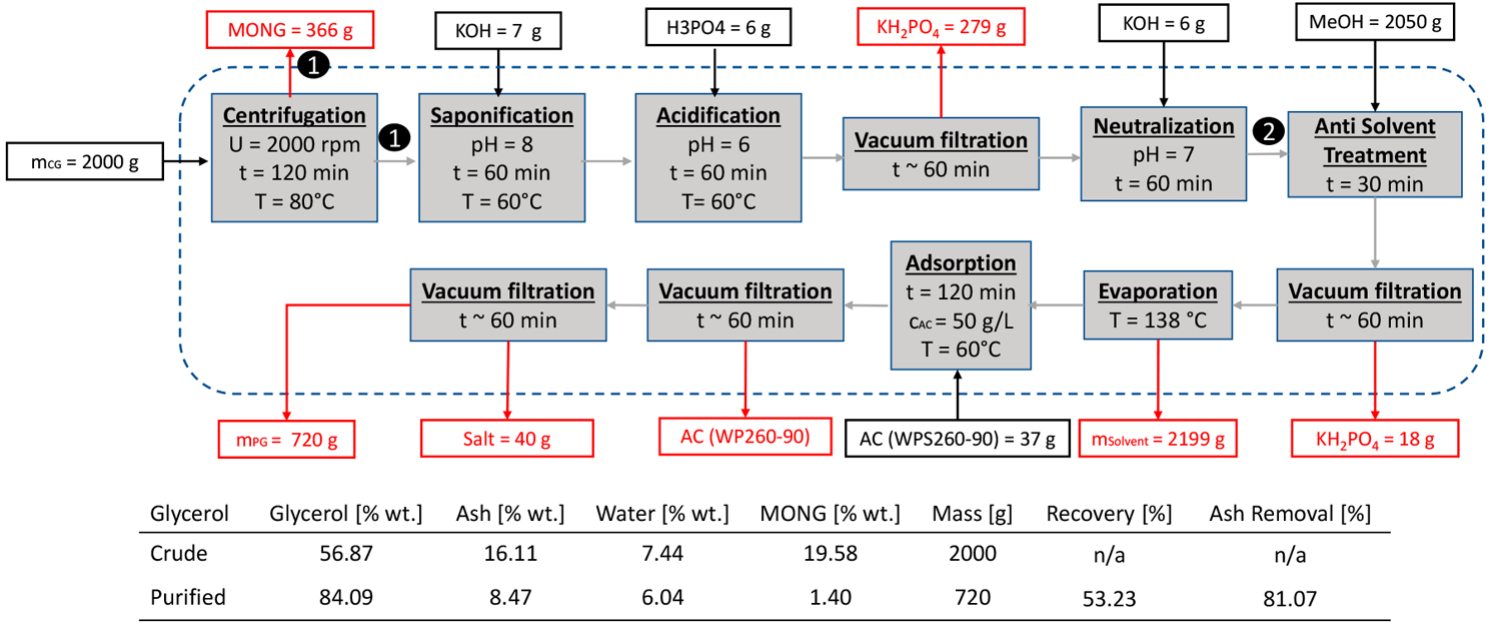
Run 1.2. Alternative purification route for STANLOW (1) with an added separation step at the beginning including detailed mass balance and compositions of crude and purified glycerol. The black dots denote sample analysis at this specific point.

In the second trial, the dark-brown, semisolid, organic layer was separated before starting the same purification steps by centrifuging the mixture for 2 h at 80 °C and 2000 rpm to break the emulsion. Afterward, the mixture was left for overnight separation. The resulting flowsheet can be seen in Figure 6.

**Figure 7:**
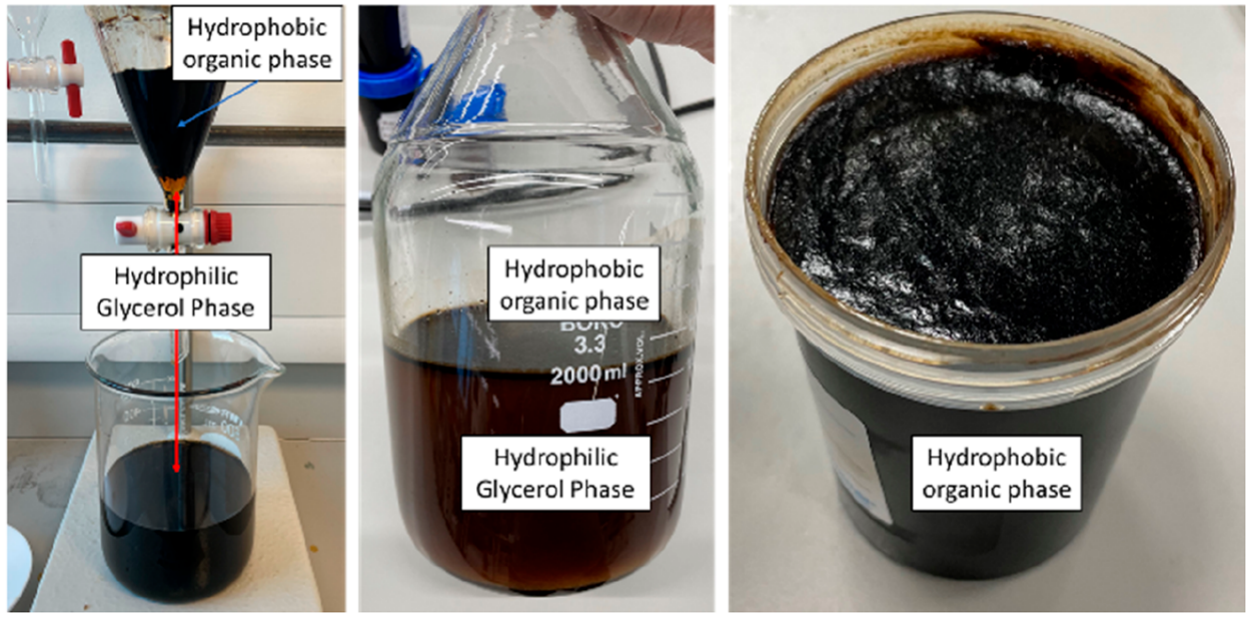
Separation of the hydrophobic organic layer. The separation between the black organic phase (top) and orange glycerol phase (bottom) is visible (left and middle). The viscous, almost solid, hydrophobic organic layer is visible after the separation (right).

The pretreatment centrifugation step shows that 366 g of material can be separated by decantation via this method, which is approximately 18% of the entire mass, as it accumulated at the top of the liquid mixture until the next day, which can be seen in Figure 7.

Compared to the results in ref (24), the experiment on a larger scale exhibits a reduced amount of chemicals (KOH, H_3_PO_4_, CH_3_OH, and activated carbon) and a slightly higher amount of salts precipitated. The sample point and organic layer purity (MONG) are also reported in Table 9. It shows that the removal of the organic layer increases the glycerol purity by 3 wt % and reduces the ash content by 2 wt % and the total ash content by 18.9%. However, it must be kept in mind that by applying this step the glycerol recovery decreases because 213.09 g out of 1017.80 g of glycerol is lost during the centrifugation step. The subsequent steps until the neutralization reaction increase the purity slightly to more to 67.5%. Furthermore, it notably reduces the ash content by approximately 4 wt % as recorded in run 1.1.

**Table 9:** Composition of the Mixture at Both Sample Points and of the Separated Organic Layer

**Composition**	**Initial (1.2)**	**Run 1.2 POINT 1**	**Run 1.2 POINT 2**	**Run 1.2 Organic Layer/MONG**	**Purified Glycerol**
Glycerol [wt %]	50.89	65.51	67.46	58.22	84.09
Ash [wt %]	16.11	14.11	10.11	16.63	8.47
Water [wt %]	7.44	6.62	4.68	5.52	6.04
MONG [wt %]	25.56	13.76	17.75	19. 63	1.40

#### 3.1.2 Characterization of Feedstock STANLOW (2)

**Figure 8:**
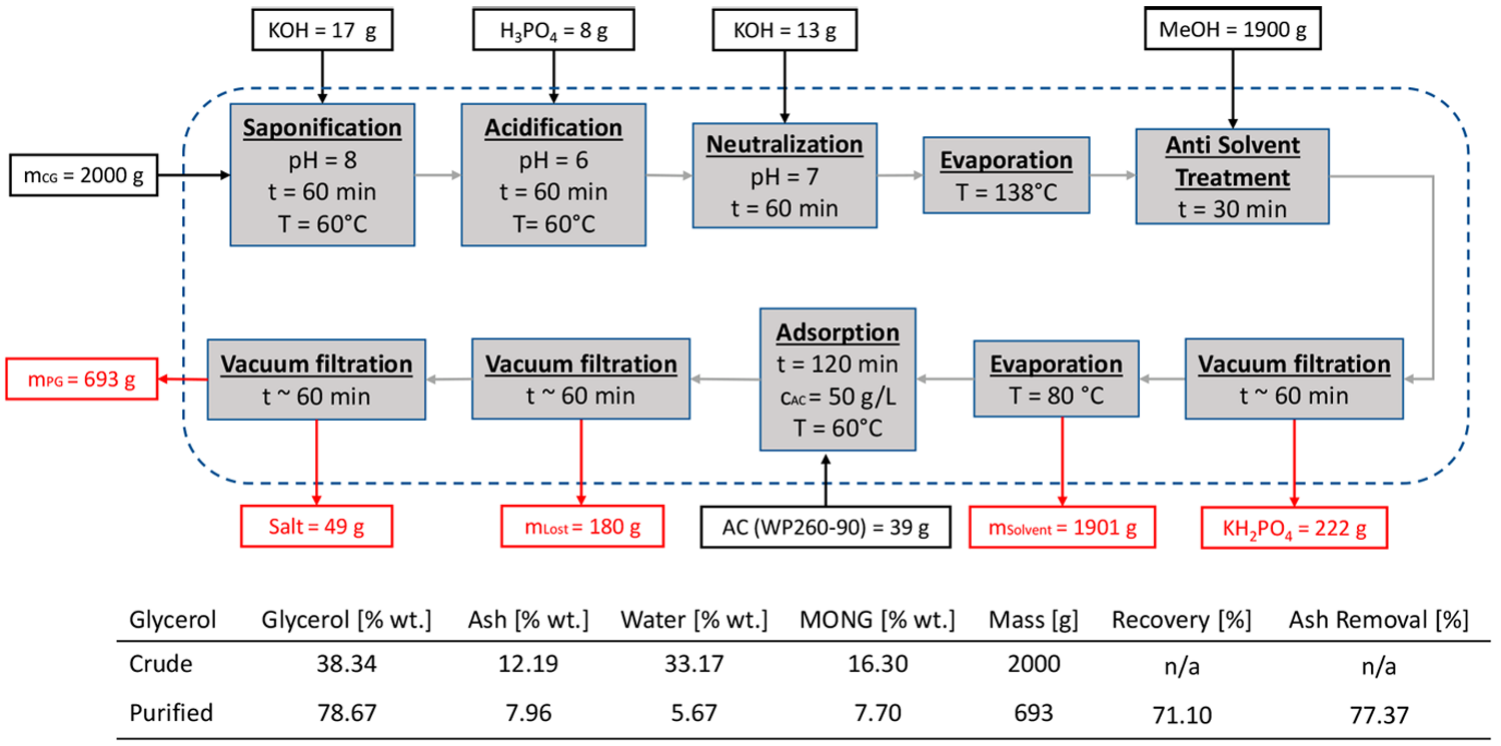
Run 2. Purification route for STANLOW (2) with an added evaporation step, including detailed mass balance and compositions of crude and purified glycerol.

STANLOW (2) is characterized by high moisture content (33.17 wt %) which makes an evaporation step necessary to reduce the methanol consumption for the antisolvent treatment. Furthermore, after acidification and overnight separation, no precipitation of salts could be observed. This is due to the high solubility of the dissolved salts in the aqueous phase. Hence, the antisolvent treatment step becomes more important to reducing the salt content. The resulting flowsheet and mass balance are reported in Figure 8.

**Figure 9:**
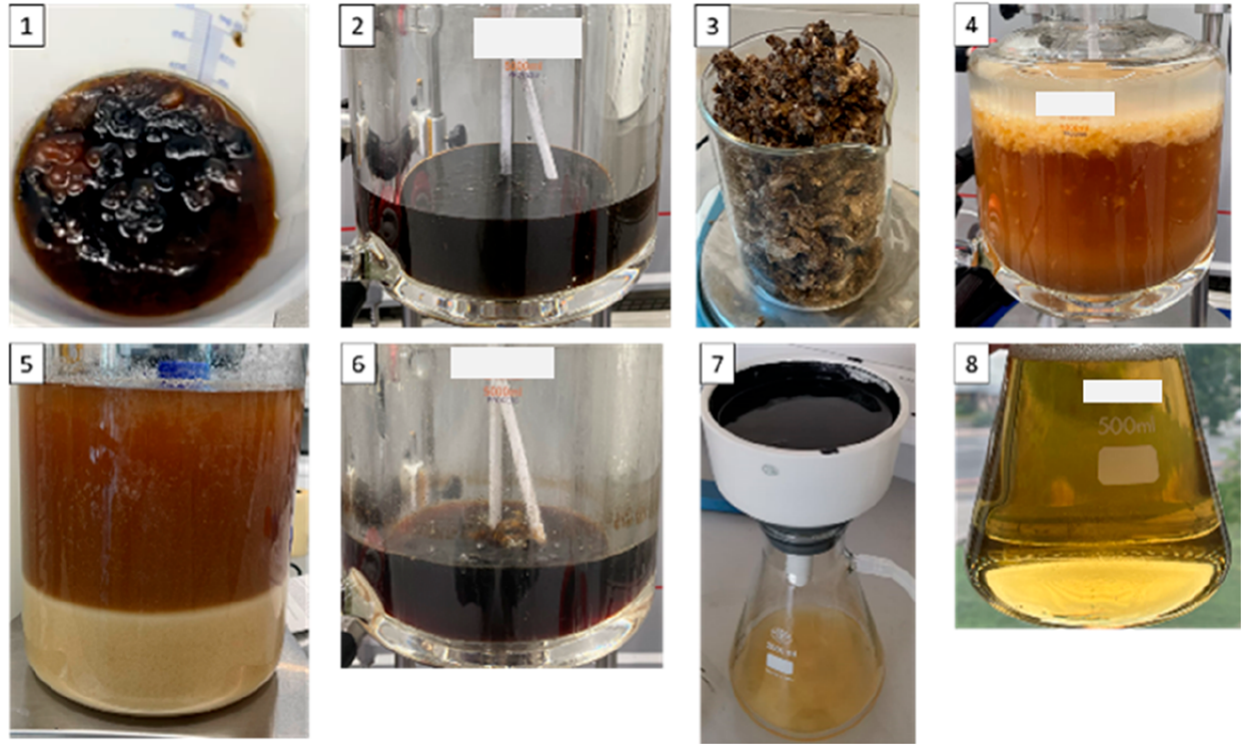
Entire process from crude glycerol to purified glycerol. (1) Crude glycerol, (2) after acidification, (3) slurry salts, (4) during AST, (5) precipitated salts settled after AST, (6) after evaporation, (7) vacuum filtration to remove AC, and (8) purified glycerol.

The major differences with STANLOW (1) in the mass balance are the use of chemicals and the negligible amount of precipitated salt acidification and neutralization steps. After the moisture content was removed, the antisolvent treatment occurred with methanol. Methanol acts as a purifying agent, yielding clear salts phase which is not soaked with MONG content. The different steps in the purification process are presented in Figure 9.

#### 3.1.3 Comparison of Purification Results for the Scaled-Up and Laboratory-Based Process

**Figure 10:**
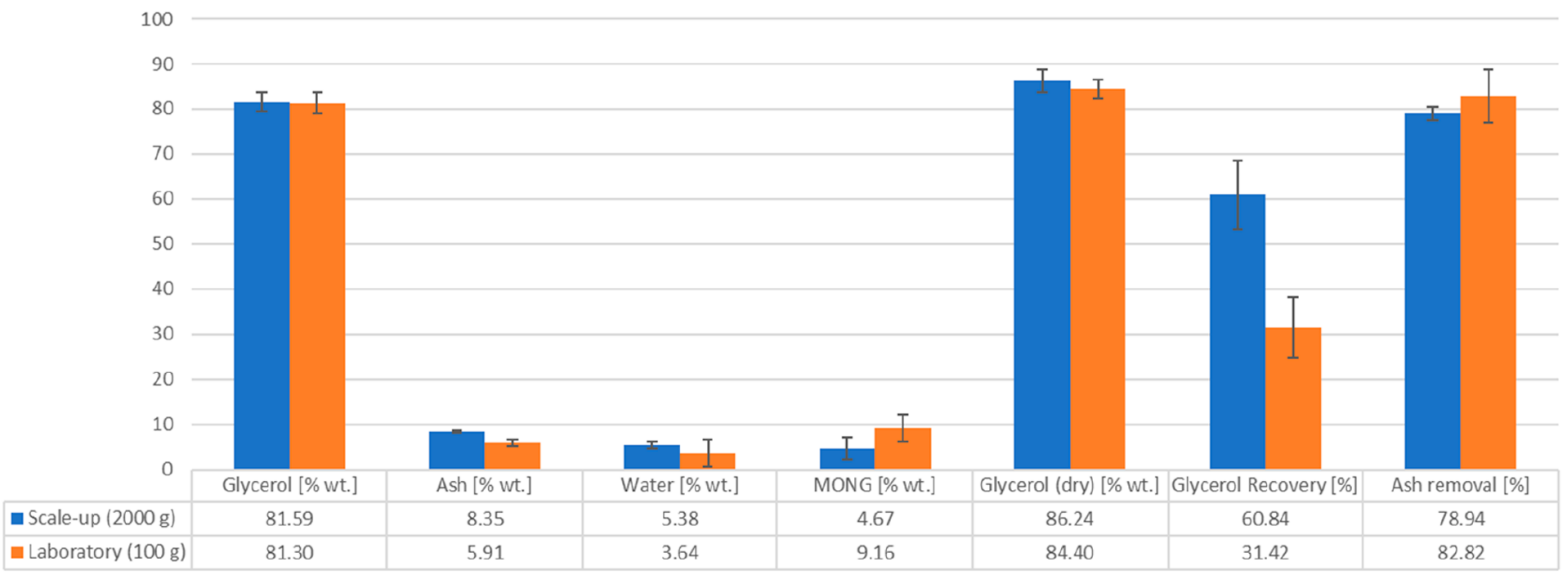
Comparison of final compositions, glycerol recoveries, and ash removal for 2000 and 100 g scales.^24^

The final purities for the scaled-up crude glycerol runs using 2000 g of starting material in comparison to the laboratory-based results using 100 g with the average results and average difference can be seen in Figure 10.

The glycerol recovery exceeds 60% with an average increase of glycerol recovery of 30% relative to that of the laboratory-based process, while the ash removal is on average 78.94 ± 1.56%, yielding a slightly lower value of 3.88% compared to that in the laboratory-based process. This is due to the weaker antisolvent properties of methanol compared to 2-propanol which was used in the laboratory-based process. At the same time, the glycerol purity is the same, confirming the effectiveness and reliability of the process regardless of the scale. The water content differs slightly, which is most likely due to the hygroscopic characteristics of the glycerol. The resulting MONG content variation is due to the ash and water contents and can be considered unvaried. The scale-up shows a significantly positive trend toward glycerol recovery while simultaneously maintaining similar purities and a slight reduction in ash removal rates.

**Figure 11:**
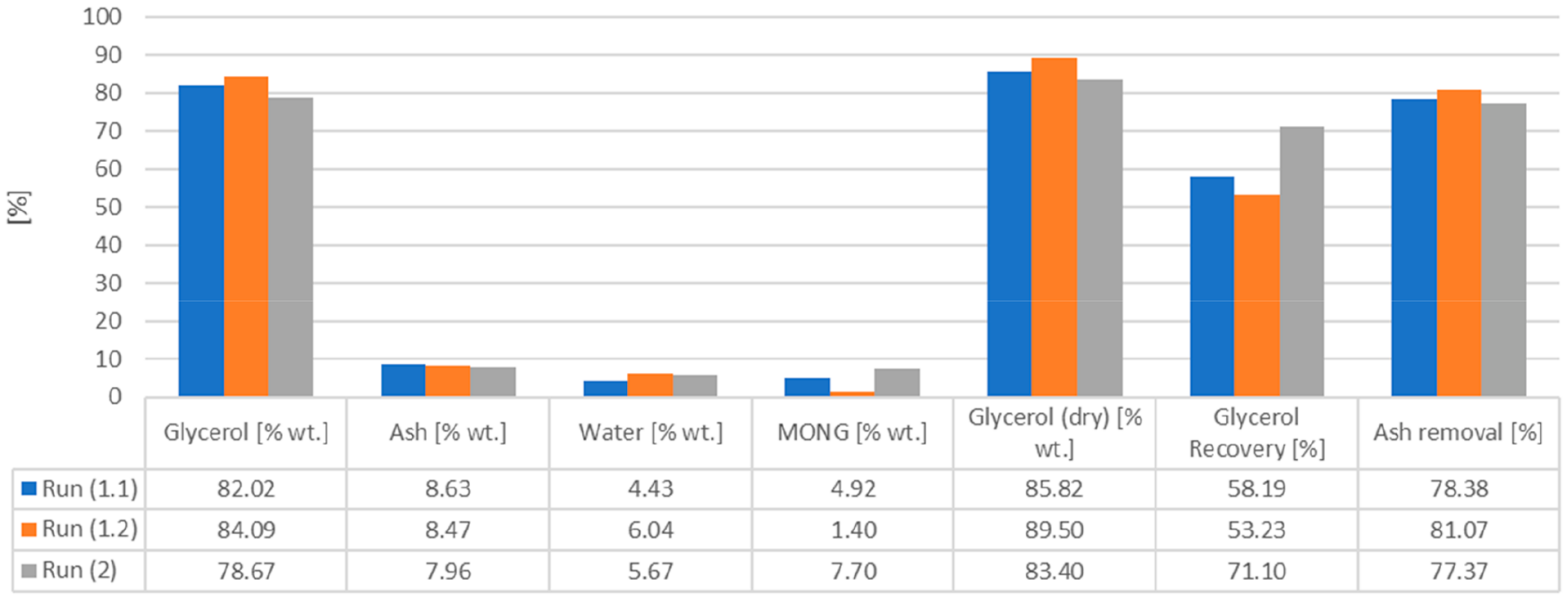
Purities, recoveries, and ash removal rates for the experiments.

Most of the KPIs reported for the three runs in Figure 11 are all in a similar range, with just very minor deviations. The main difference involves the glycerol recoveries and purities. The glycerol recovery for run 1.2 is lower due to the negative effect of a centrifugation step; on the other hand, the centrifugation step increases the (dry) glycerol purity by 3.7%. The differences in glycerol purities for runs 1.1 and 1.2 are explained by the centrifugation step, and that for run 2 is due to the initial feedstock composition.

The glycerol recovery of run 2 is higher than that of runs 1.1–1.2. The reason for this is due to the inherent difference in the feedstock composition (with and without moisture). The variation in dry glycerol purity is mainly given by the existence of different MONG contents present in the two different feedstocks.

However, the process shows significant consistencies for different feedstocks derived from industrial refineries.

The process yields on average a glycerol dry purity of approximately 86.24 ± 2.51%, which renders it suitable for use in energy applications or ready for a deeper purification with alternative methods. The lower purity compared to that in previous work in the literature depends on the following: (i) the crude glycerol used in this process that has already undergone significant post-treatment steps at the refinery premises to recover valuable FFA, MeOH, and salts; therefore, different chemical agents have been added to the mixture, making it more impure; (ii) glycerol is derived exclusively from waste feedstocks such as animal fats, tallow, POME, or material from the sewers; therefore, the biodiesel production yields several solid byproducts; and (iii) the process developed in this work is carried out under milder conditions in terms of acidification/neutralization and process conditions (e.g., temperature and pressure) to be relevant for industrial use and therefore reduce the cost for implementation in the short term.

### 3.2 Technoeconomic Assessment

In this section, the overall key performance indicators of implementing the flowsheet developed here are calculated to assess the feasibility of implementing such a process at scale.

**Figure 12:**
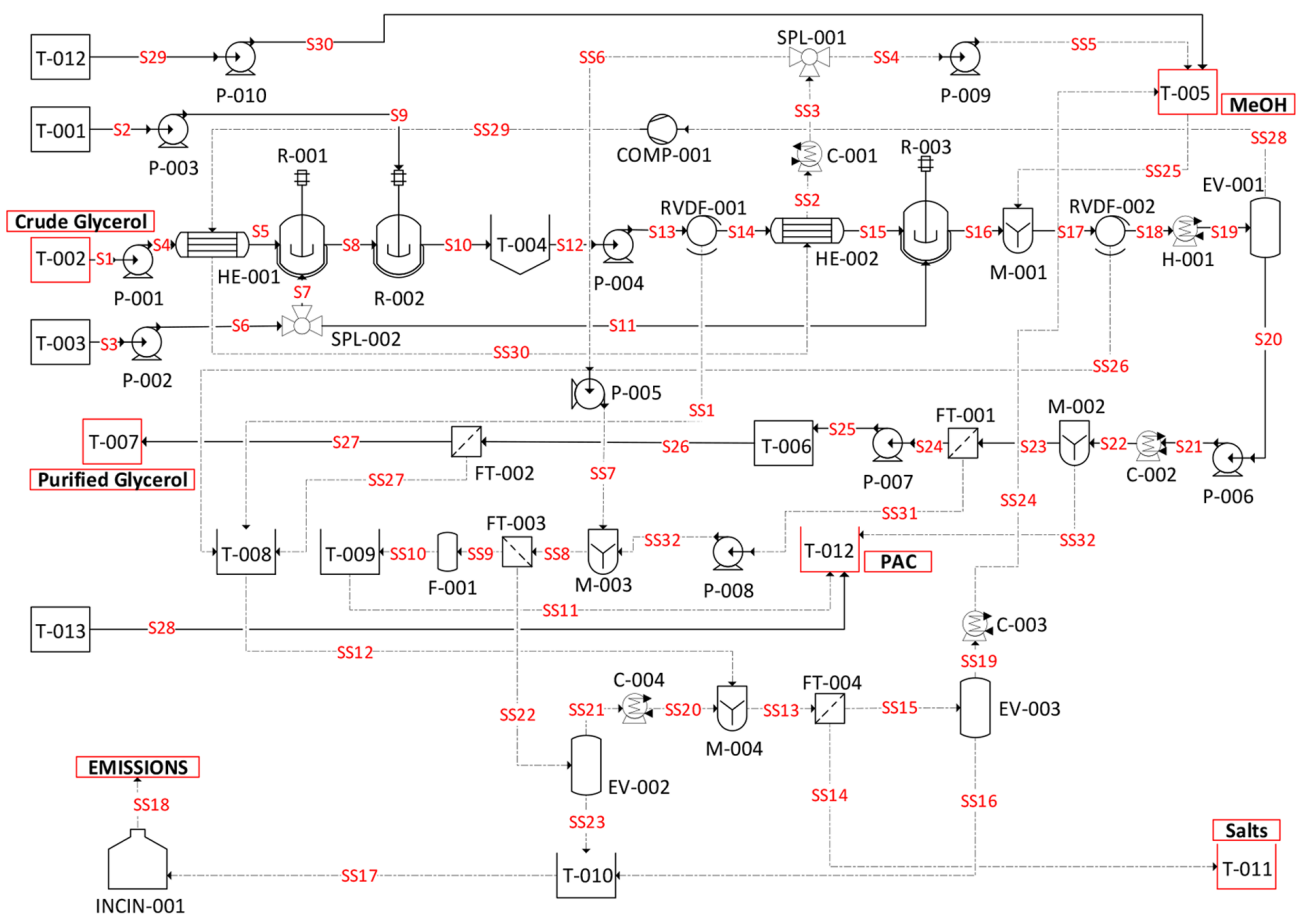
Process flow diagram of the crude glycerol purification process (S, stream; SS, substream).

The process flow diagram (PFD) has been developed following the experimental study reported here and scaled up to an industrial case, which could be operated under the conditions tested at the laboratory scale. The crude glycerol purification derived from the Aspen Plus flowsheet can be seen in Figure 12, with the detailed design and cost of each piece of equipment reported in the Supporting Information. The process is generally based on run 1.1, which means that a separate stream of MONG is not considered in this process after acidification. Additional steps to increase the efficiency of the process and reduce the waste of the reagents are included to provide a realistic design which is also profitable.

The inlet stream compositions are shown in Table 10. The detailed compositions and thermodynamic conditions of *T*, *p*, etc. of each stream (S) and substream (SS) can be found in the Supporting Information “M&H Final”.

**Table 10:** Inlet Composition Streams for the Simulation

**Property**	**Crude Glycerol [S1]**	**KOH [S7]**	**KOH [S11]**	**H**_**3**_**PO**_**4**_**[S2]**	**PAC [S28]**	**METHANOL [S29]**
Molar flow [kmol/h]	43.63	0.64	0.52	0.19	0.77	3.90
Mass flow [kg/h]	2829.16	14	11.2	11.2	9.20	125.04

**Component Mass Fraction [wt %]**						
Glycerol	61.86	0	0	0	0	0
Methanol	0	0	0	0	0	100
Ash	16.87	33.2	33.2	85	0	0
Water	7.41	52.35	52.35	15	0	0
MONG	13.86	14.45	14.45	0	100	0
Note	Feedstock	12.5 M KOH	12.5 M KOH	85% H_3_PO_4_	Carbon	Fresh stream

The crude glycerol (S1) is first pumped (P-001), heated to 60 °C in a heat exchanger (HE-001) to decrease the viscosity, and then pumped into the saponification reactor (R-001). After saponification, the material is discharged to the acidification reactor (R-002), followed by an overnight separation in a settling tank (T-004). The slurry is then pumped (P-004) through a rotary vacuum drum filter (RVDF-001) where the salts are collected in a separate container (T-008) and the liquid is heated to 60 °C (HE-002) and directed to the neutralization reactor (R-003). Afterward, the liquid is mixed with methanol from a separate tank (T-005) and redirected into a rotary vacuum drum filter (RVDF-002) to obtain the relevant salts, which are collected in a separate container (T-008). The mixture is then heated to approximately 140 °C (heater H-001) and separated in an evaporator (EV-001) to recycle the methanol. The vapor-phase methanol is led through a blower and two heat exchangers (HE-001 and HE-002) and further condensed in the cooler (C-002). The methanol stream is then split (SPL-001) and recycled into the process through the tank (T-005) and a bleed stream that is pumped (P-005) to a mixer (M-003) where it is used to purify the spent activated carbon. The hot liquid outlet of the evaporator (EV-001) is cooled (C-002) and mixed with powdered activated carbon (0.05 kg_PAC_/kg_glycerol_ (M-002)) and then led into a filter (FT-001) to remove the powdered activated carbon. The obtained liquid after filtration (FT-001) of the activated carbon is now stored in a tank (T-006), where it is kept for 2 days before some additional salts are precipitated, which are removed via filtration (FT-002). The separated salts are stored in a container (T-009) while the final product is sent to a tank (T-007).

The spent activated carbon (SS31) is mixed with methanol (SS7) in a ratio of 1.67 kg_PAC_/kg_MeOH_ in a mixer (M-003) to extract polar impurities and then filtered (FT-003), where the activated carbon is now regenerated in a furnace (F-001), stored in a container (T-0091), and redirected into the PAC tank (T-012). The obtained stream (SS22) from the filtration (FT-003) is led into an evaporator operated at 57 °C (EV-002). The resulting methanol-rich vapor stream (SS21) is later condensed at 25 °C (C-004) and then mixed (M-004) with the salts to remove organic impurities from them (SS13). Afterward, the salts are separated via filtration (FT-004) and stored in a tank (T-011) while the liquid obtained is again evaporated at 95 °C (EV-003) to obtain a methanol-rich vapor phase which is again condensed (C-003) and redirected into the process (T-005). The liquid phase of both evaporator outlets (EV-002 and EV-003) are waste streams (SS23 and SS16) containing unreacted glycerol, FFAs, and methanol, with impurities which are incinerated (INCIN-001). The generated heat is used in the process.

The outlet stream compositions for the simulated PFD are listed in Table 11.

**Table 11:** Composition of the Solid/Liquid Streams

**Property**	**Purified Glycerol [S27]**	**Waste to INCIN [SS17]**	**Salts [SS14]**
Molar flow [kmol/h]	23.61	241.36	2.05
Mass flow [kg/h]	1630.87	6228.46	314.32

**Component Mass Fraction [wt %]**	**Purified Glycerol [S27]**	**Waste to INCIN [SS17]**	**Salts [SS14]**
Glycerol	82.22	0	0
Methanol	0	0	0
Ash	8.68	0.23	100
Water	4.6	9	0
MONG	4.5	90.29	0
Remarks	Product	Waste	Byproduct

A glycerol recovery of 76.62% is achieved with the process. An improvement of approximately 20% can be observed compared to the scale, which is reasonable considering that the scale effect also appreciated in the experimental campaign from 100 to 2000 g. Simultaneously, the ash content reduction is approximately 70.34%, which is slightly less than 76.40 ± 0.06%, confirming the same trend. Looking at the energy requirement (Table 12), the process has almost no heat consumption as the boiler used for waste stream combustion can be used to generate LP steam at 6 bar, 160 °C for the evaporator and distributed in the plant. The combustion generates 0.70 tons of CO_2_ per ton of purified glycerol. It must be noted that this emission is for about 85% of biogenic origins since the carbon source is glycerol or MONG, which has not been separated. The remaining 15% is methanol (15%), used as a solvent, which is not recovered, and it is assumed to be produced from fossil fuels.

**Table 12:** Energy Balance and CO_2_ Emission of the Purification Process

**Process**	
Glycerol recovery [%]	76.62
Ash removal [%]	70.34

**Utilities**	
Heat produced from combustion (INCIN-001) [kW]	3145.81
Heat required for evaporator (EV-001, EV-002, EV003, H1) [kW]	–3115.66
Electricity process pumps/blowers [kW]	131.93
Electricity to circulate cooling [kW]	91.48
Cooling water [m^3^/h]	262.24

**Emission**	
Solid waste (ashes) [kg/h]	211.53
CO_2_ emissions [kg/h]	1145.45
CO_2_ per kg of product – biogenic [g_CO_2__/kg_PG_]	596.05
CO_2_ per kg product – methanol [g_CO_2__/kg_PG_]	106.31

**Specific Consumption**	
Specific consumption of total electricity [kWh_el_/m^3^_PG_]	178.726
Specific consumption of heat [kWh_heat_/m^3^_PG_]	0.02
Specific consumption of methanol [kg_meoh_/m^3^_PG_]	100.03
Specific consumption of activated carbon [kg_AC_/m^3^_PG_]	7.36
Specific consumption of 12.5 M KOH [kg_KOH_/m^3^_PG_]	20.16
Specific consumption of 85% H_3_PO_4_ [kg_H_3_PO_4__/m^3^_PG_]	8.96

In terms of specific consumptions of chemicals, the biggest contribution is due to the consumption of methanol by 100 kg_MeOH_ per m^3^ of purified glycerol. The electricity demand comes mainly from the pumps, and a blower and makes up most of the consumption (136.9 kWh_el_/ton_PG_). As a comparative example, the study conducted by Braga et al.^22^ presented a heat demand of 166.5 kWh_th_/ton_PG_ at the reboiler of the vacuum distillation column, thus remarkably higher while the electricity demand was not reported. From a more industrial perspective, Argent Energy is currently planning the construction of a vacuum distillation refinery with pretreatment facilities with a specific electricity and heat consumption of approximately 1000 kWh/ton_PG_ for a plant of 50,000 ton/y of glycerol quality of at least 99.7%.^36^

Full details of M&H balances, stream tables with material properties and composition, and cost analysis for each piece of equipment is reported in the Supporting Information. A full disclosure of the economic model is reported in the same file.

The total investment cost is estimated to be 19.15 M€. In terms of equipment, the main costs are associated with reactors, tanks, and the furnace.

In terms of operating costs, the main terms to be considered are methanol (0.40 M€/y) and electricity (0.32 M€/y, given the high cost considered). Methanol is assumed to be purchased from suppliers (conservative assumption); however, its use and cost could be reduced by recycling part of the waste stream in the biodiesel plant, thus partially reducing the total cost of production. Since the waste glycerol is not sent for disposal, the proposed process provides a saving of 3.39 M€/y compared to the current costs incurred by biodiesel producers and the production of byproduct fertilizer yield of 0.37 M€/y. The final cost of purified glycerol is 19.2 €/ton. Such cost makes waste glycerol very competitive in terms of energy feedstock for other processes such as reforming/gasification^37^ or other added value chemicals^38^ or livestock feeding.^39^

**Table 13:** Detailed Investment Cost

**Investment Cost**	
ISBL cost [M€]	5.31
Pumps	4.40%
Heat exchangers	3.04%
Tanks	17.43%
Reactors	21.87%
Rotary filters	11.83%
Mixers	0.35%
Furnace	19.12%
Heater/cooler	7.90%
Evaporators	4.57%
Compressor	4.43%
Balance of plant	5.08%
OSBL [M€]	2.13
EPC [M€]	1.86
Contingencies [M€]	0.74
Total plant cost [M€] @2010	10.05
Total plant cost [M€] @2022 in The Netherlands	18.24
Working capital [M€]	0.91
Total investment cost [M€]	19.15

Comparing the operating costs from Table 14 with those from Chol et al.^8^ who calculated a cost of $50.45 per kg of purified crude glycerol, it is clear that the costs for the purification in this process are much cheaper. In the results presented by Braga et al.,^22^ the costs are significantly lower (they estimated OPEX of 886 € per tonne of purified product). However, in both studies, glycerol is produced at a higher purity. While Braga et al.^22^ used vacuum distillation (starting with 3 wt % ashes), Chol et al.^8^ used membrane filtration, which adds significant cost to the purification process but also allows for higher purity (starting with 4.9 wt % ashes). However, those processes are not suitable for this process in which the ash content is above 16 wt %. In the case of CAPEX, the process here is higher than those in the literature because of the higher complexity and level of detail in the design proposed.

**Table 14:** Operating Cost

**Operating Cost of Production**	
Variable operating cost [M€/y]	**–2.86**
Raw material	–3.39
Byproduct	–0.37
Utilities	0.40
Consumables	0.59
Fixed operating costs [M€/y]	**1.88**
Operating labor	0.60
Supervision	0.15
Direct salary overhead	0.24
Maintenance	0.18
Property taxes	0.08
Insurance	0.08
Rent of land	0.08
General and administration costs	0.39
Allocated environmental charges	0.08
Annual capital charge [M€/y]	1.23
Total annual cost of production [M€/y]	**0.25**
*Cost of the product [€/ton]*	**19.19**

## 4 Conclusions

The purification of end-of-life glycerol was scaled up to 2000 g using a physiochemical process. The scaled-up purification process delivers an average glycerol purity of 81.6% with an average ash content of 8.3 wt % as well as an average glycerol recovery of 61.7 wt % and an ash removal rate of 78.9 wt %. Water removal is needed in the case of glycerol with high moisture content. The treatment of different samples exhibited minor differences in the final quality of the purified glycerol, providing consistency and robust validation of the purification methods and sequence developed. The assessment at the industrial scale resulted in 76.6% recovery with the same purity and an overall ash removal rate of 70%. The energy requirement is primarily associated with the electricity for pumps and blowers (137 kWh/ton of purified glycerol), while no heat is required after thermal integration between units. Overall, the glycerol of cost production is limited to 19.2 €/ton given the cost savings from not disposing of the crude feedstock. While the level of impurity will not be acceptable for some processes,^40−42^ low-cost purified glycerol could be of interest to produce added-value chemicals, hydrogen, or additional biofuels.

This work confirmed the soundness and viability for physiochemical purification of second-generation byproduct waste glycerol providing a comprehensive industrial perspective in terms of scaled-up pathways and performance. These results provide more confidence to industry and end-users on the opportunity for waste valorization. The flexibility of feedstock quality is promising for applying the same purification sequence with other waste materials such as sludges and wastewater, which are currently disposed of at cost. Such achievement is aligned with the utmost need to develop more environmentally friendly and cost-effective chemical and biochemical processes.

## Supporting Information Available

Heat and material balance of process; costing model for CAPEX and OPEX calculations (XLSX)

## List of Abbreviations and Definitions

ACS: American Chemical Society
ANOVA: Analysis of Variance
AOCS: American Oil Chemists’ Society
BS: British Standard
CAGR: Compound Annual Growth Rate
DOE: Design of Experiment
GC–MS: Gas Chromatography–Mass Spectrometry
FAME: Fatty Acid Methyl Ester
FFA: Free Fatty Acids
FOG: Fats, Oil, and Greases
HPLC: High-Performance Liquid Chromatography
ICP–OES: Inductively Coupled Plasma–Optical Emission Spectrometry
MONG: Matter Organic Non-Glycerol
PAC: Powdered Activated Carbon
POME: Palm Oil Mill Effluent
RID: Refractive Index Detector
RSM: Response Surface Methodology
TAG: Triacyl-glyceride
UCO: Used Cooking Oil
